# Patients’ Experiences of Person-Centered Care in the Context of Allogenic Stem Cell Transplantation

**DOI:** 10.1177/10547738241302393

**Published:** 2024-12-12

**Authors:** Anna O’Sullivan, Carina Lundh Hagelin, Katarina Holmberg, Karin Bergkvist, Sidona-Valentina Bala, Yvonne Wengström, Annika Malmborg Kisch, Jeanette Winterling

**Affiliations:** 1 Marie Cederschiöld University, Stockholm, Sweden; 2 Sophiahemmet University, Stockholm, Sweden; 3 Karolinska Institutet, Stockholm, Sweden; 4 Uppsala University, Uppsala, Sweden; 5 Section of Rheumatology, Helsingborg Central Hospital, Sweden; 6 Lund University, Sweden; 7 Karolinska University Hospital, Stockholm, Sweden; 8 Skåne University Hospital, Lund, Sweden

**Keywords:** allogeneic hematopoietic stem cell transplantation, cross-sectional study, person-centered care, patients’ experiences

## Abstract

Studies addressing patients’ experiences of person-centered care (PCC) in the context of allogeneic hematopoietic stem cell transplantation (allo-HSCT) are scarce; hence, this study aimed to explore patients’ experiences of PCC, and its associations with individual characteristics and health-related quality of life, in the context of allogeneic stem cell transplantation. It is a cross-sectional survey study, in patients who had undergone an allo-HSCT at one center in Sweden. The PCC instrument for outpatient care in rheumatology (PCCoc/rheum) was used. Descriptive and analytical statistics were employed. The study had 126 participants, evenly distributed males and females, 18–79 years old (>60% were 50–69 years old), and most were (>70%) married or cohabiting. The sum score for all items on PCCoc/rheum ranged from 20 to 72 (higher score = higher degree of PCC), with a mean value of 62.67 (SD: 9.863). Most participants (87–99%) agreed with the level of person-centeredness for 22 of the 24 items. Of the participants, 83.3% agreed that they had undisturbed conversations, that their problems had been taken seriously (79.0%), that they had an opportunity to tell their story (77.8%), and collaboration with the nurse was good (77.6%). A sizeable proportion disagreed that the care environment was welcoming (11.9%), family members’ involvement (13.7%), and the possibility to influence the care (15.5%). The fulfillment of PCC was rated as high, but the results indicate that there is room for improvement regarding the possibility of influencing the care and family members’ involvement.

## Background

An increase in the number of allogeneic hematopoietic stem cell transplantations (allo-HSCT) has been seen over the last decade, with more than 80,000 procedures performed internationally each year, and this is estimated to reach 1 million allo-HSCTs by the end of 2024 (Rocha et al., 2021). Allo-HSCT is an advanced medical treatment aiming to cure a fatal diagnosis, commonly hematological malignancies, requiring an individually matched donor. Approximately 300 transplantations are performed yearly at six transplant centers in Sweden (Passweg et al., 2019). The treatment trajectory mainly includes three phases, starting with 4–6 weeks of hospitalization, mostly in isolation due to the intensive treatments and high risk of infections. Discharge is followed by intensive post-discharge care and a long period of rehabilitation. Although the goal of an allo-HSCT is to cure the disease, the outcome of the treatment is often uncertain, and medical complications are common, for example, infections and the immune reaction graft versus host disease (GvHD) (Sureda et al., 2015). Patients experience physical and psychological symptoms as well as social and existential distress during the entire trajectory (Eriksson et al., 2023; Kuba et al., 2017). Post-allo-HSCT patients experience functional impairment as well as psychological distress related to an uncertain future with a risk of relapse and complications (Braamse et al., 2013). Fatigue post-allo-HSCT has been shown to impair patients’ social adjustment after transplantation (Park et al., 2019). The social network (i.e., the healthcare team, family, and friends) is vital in the care of allo-HSCT patients (Bergkvist et al., 2018) and important for social adjustment post-allo-HSCT (Park et al., 2019).

Person-centeredness and person-centered care (PCC) are important emerging perspectives in health care that acknowledge the person in need of care (Edvardsson et al., 2008; Öhlén et al., 2019). PCC is a philosophical approach to healthcare on which the healthcare system must be based and where the person is central (McCormack & McCance, 2006, 2016). In PCC, the person is not just their illness; this type of care considers the person’s resources, conditions, and obstacles. Respect for the person’s integrity and mutual security, understanding, and trust in the care relationship are key. Previous studies have shown a lower reported HRQoL post-allo-HSCT (El-Jawahri et al., 2016; Eriksson et al., 2023) associated with higher age (Eriksson et al., 2023), fewer socioeconomic resources (Mosher et al., 2011), and higher pre-HSCT perceptions of social support (Amonoo et al., 2021). PCC has been found to improve patients’ health-related quality of life (Brännström & Boman, 2014; Pirhonen et al., 2017), outcomes, preparedness for treatment, and safety, as well as the quality and safety of hospital care (Harding et al., 2015; Öhlén et al., 2019; Rose & Yates, 2015). Furthermore, PCC has proved especially effective in care after acute coronary syndrome for patients with lower educational attainment (Fors et al., 2016).

There are several existing instruments to measure PCC (De Silva, 2014) and a great variety of how PCC is measured by different tools (Louw et al., 2020). A review study showed that the conceptual underpinnings of the tools are rarely explicit or the tools have not been applied in actual research since being developed (Edvardsson & Innes, 2010). PCC interventions need to be contextualized to specific patient populations and treatment paths, although there are universal aspects of PCC applicable across healthcare services (Ekman et al., 2015). Therefore, there is a need to provide insights into practice and identify areas for improvement of PCC, for which measuring instruments may be important. Despite the highly individualized medical treatment in allo-HSCT, there is scarce knowledge about patients’ experiences of PCC in this setting. Therefore, this study aimed to explore patients’ experiences of PCC and its associations with individual characteristics and HRQoL, in the context of allogeneic stem cell transplantation.

## Methods

### Design

This study employed a cross-sectional survey design with descriptive and analytical statistics for data analyses.

### Setting and sample

At the allo-HSCT center in Stockholm, about 100 patients are transplanted each year. The ward is staffed by registered nurses, assistant nurses, and physicians at all times, with access to physiotherapists, dieticians, and counselors if needed. The inclusion criteria for the study were as follows: adults (≥18 years old), who had undergone an allo-HSCT 12–36 months prior (2017–2020) at the center in Stockholm, and who spoke and understood Swedish. Exclusion criteria were people with cognitive impairment, those in need of support from someone else with self-care, or those with acute illness who are too ill to participate.

### Recruitment and data collection

Recruitment was carried out with the assistance of a head of nursing development at the center. An invitation to participate in the study was sent to all eligible participants by post with two reminders. The invitation contained a link to the survey; information about the study, including researchers’ contact information; and information stating that the study was performed in cooperation with the hospital in which they had their allo-HSCT. The written information assured confidentiality and the right to withdraw from the study at any time without explanation. A filled-out online instrument or returned postal one was considered as consent to participate.

### Measures

Patients’ experiences of PCC in connection to their transplantation were assessed using the Person-Centered Care instrument for outpatient care in rheumatology (PCCoc/rheum) by Bala, Forslind, Fridlund, Samuelson, et al. (2018) and has previously been used in a study on outpatients with inflammatory arthritis (Van Slingerland et al., 2022). A conceptual framework for operationalizing PCCoc/rheum was developed based on the experiences of people with rheumatoid arthritis (RA), person-centeredness principles, and existing PCC frameworks. The framework comprises five main domains: *Social environment (SoE)* includes how people are confirmed, received, approached, and communicated with; conditions for good relationships and for establishing a friendly atmosphere; and the physical care environment. *Personalisation (P)* involves the identification and recognition of each person’s needs, concerns, preferences, and values, as well as their abilities and capabilities. *Shared decision-making (SDM)* refers to collaboration between the person and the nurse, including understanding the person’s situation; agreement about care needs; planning and coordination of care; and communication between healthcare professionals as well as family members. *Empowerment (E)* represents the enabling of individuals’ resources and abilities. *Communication (C)* refers to the exchange of information; management of emotions and feelings; and the relationship between the nurse and person; hence, the domain communication is considered to apply to all of the PCCoc/rheum items. The instrument contains 24 statements posed from the participants’ perspective—with personal pronouns such as I, my, and me—which participants were asked to rate on a 4-point scale (0–3) ranging from “totally disagree” to “completely agree” (Bala, Forslind, Fridlund, Samuelson, et al., 2018). PCCoc/rheum was chosen as it is a validated Swedish instrument that focuses on nursing care. Furthermore, PCCoc/rheum suited the patient group investigated in this study and also reflected central ideas in a model for PCC during allo-HSCT developed by the research group which is currently tested in allo-HSCT clinics.

In addition, patients’ characteristics were collected including age, sex, educational attainment, having children living at home, marital status, and whether the allo-HSCT had occurred during the COVID-19 pandemic or before. HRQoL was assessed using the Global Health Scale from the European Organization for Research and Treatment of Cancer Quality of Life Questionnaire Core-30 (EORTC QLO C-30). In total, the scale includes 30 items, and the two final questions rated on a 7-point Likert scale of 1 (very poor) to 7 (excellent) yield a global health sum score together ranging between 0 and 100; that is, “How would you rate your overall health during the past week?” and “How would you rate your overall quality of life during the past week?”(Aaronson et al., 1993).

### Statistical analyses

Descriptive statistical analyses were used to explore the variables derived from the instrument items, participant characteristics, and HRQoL. A sum score of the PCCoc/rheum was calculated, yielding a raw total score between 0 and 72, with higher scores indicating a greater degree of perceived PCC (Bala, Forslind, Fridlund, et al., 2018).

Multiple linear regression analysis was employed to investigate associations between the sum score for PCC and participants’ age, sex, educational attainment, having children living at home, marital status, and whether transplanted during the COVID-19 pandemic. The independent variables were dichotomized for the analyses. To calculate the sum score for Global Health and PCCoc/rheum, missing data were replaced based on person-mean imputation (Bell et al., 2016), if the missing data did not exceed 20% (Downey & King, 1998). HRQoL was calculated through the global health sum score from the EORTC QLQ-C-30 in accordance with the scoring manual (Fayers et al., 1995). Variables were entered with the forced entry method, that is, all variables were entered simultaneously. A separate linear regression was performed to analyze associations between the sum score PCC and HRQoL through the global health sum score. Co-variables were considered to have a significant association with the outcome if *p* ≤ .05. For statistical computations, Statistical Package for the Social Sciences (SPSS) version 27.0 (IBM Corp., Armonk, NY, USA) was used.

## Results

### Participants

A total of 126 individuals chose to participate, representing a 60% response rate. The participants were evenly distributed into males and females, 21–75 years old (62.3% were 50–69 years old), most of whom (79.2%) were married, cohabiting, or had a partner (Table 1). Non-responders’ characteristics were not available. Overall health during the past week was reported as a mean value of 4.98 (range 1–7) and for overall quality of life during the past week, the mean value was 5.21 (range: 1 [very poor]–7 [excellent]). Most participants rated their overall health during the last week as five or above (63.1%), and 71.5% responded that their overall quality of life during the last week was a five or above. The mean sum score for current global health was 68.5 (SD: 23.7) (Table 1).

**Table 1.** Characteristics of the Participants.

Characteristics	%^a^	*n*	Missing^b^
Age (years)			4
18–29	4.1	5	
30–39	7.4	9	
40–49	11.5	14	
50–59	27.9	34	
60–69	34.4	42	
70–79	14.8	18	
Sex			1
Male	52	65	
Female	48	60	
Marital status			1
Married/partner	79.2	99	
Single*	20.8	26	
Children living at home			
Yes	29.4	37	
No	70.6	89	
Educational attainment			
Lower secondary education	7.9	10	
Secondary education	27	34	
Post-secondary education/Vocational education	18.3	23	
Higher education	46.8	59	
Years since transplantation			6
4 years	23.3	28	
3 years	24.2	29	
2 years	24.2	29	
1 year	28.3	34	
Transplantation during COVID			6
No	75.8	92	
Yes	24.2	28	
Current global health			
Mean (*SD*)	68.5 (23.7)		
Median (range)	70.9 (0–100)		

a Column percentage displayed.

b Missing cases are excluded from the analyses.

* Includes single, divorced, and widowed.

### Reported experiences of PCC

**Figure 1.**
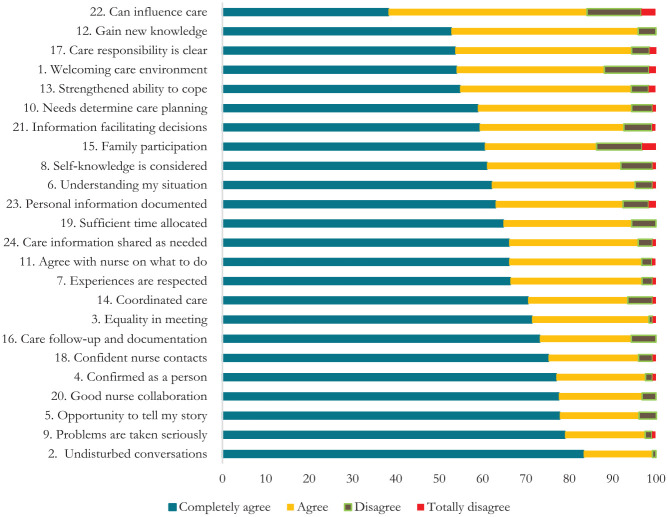
Results of all PCCoc/rheum items ordered according to the lowest perceived degree of person-centeredness. *Note*. Missing cases are excluded from the analyses. PCCoc/rheum = the Person-Centered Care instrument for outpatient care in rheumatology.

The sum score for all PCCoc/rheum items ranged between 20 and 72, with a mean value of 62.67 (SD: 9.863). About half of the participants had a sum score of 65–72. Most participants (87%–99%) completely agreed or agreed with the level of person-centeredness for 22 of the 24 items. Of the participants, 83.3% completely agreed that they had undisturbed conversations (item 2); 79.0% that their problems had been taken seriously (item 9); 77.8% reported having had an opportunity to tell their story (item 5); and 77.6% described good collaboration with the nurse (item 20). Furthermore, 77.0% said they were confirmed as a person (item 4), 71.4% experienced equality in the care meeting (item 3); and 70.5% said they had coordinated care (item 14). Eight items (1, 10, 12, 13, 15, 17, 21, and 22) had a lower degree of reported perceived PCC, with 60% or less responding that they completely agreed with a mean of 2.5 or lower (range 0–3). The three items with the lowest degree of participants responding “completely agree” were as follows: I gain new knowledge in the meeting with the nurse (52.8%), my part of the care responsibility is clear (53.7%), and I can influence my care (38.3%). Three items had a higher proportion of participants responding that they disagreed or totally disagreed: Welcoming care environment (11.9%), family participation—that my family can participate as much as I liked in my care (13.7%), and that I can influence my care (15.5%) (Table 2 and Figure 1).

**Table 2.** Participants’ Responses for All Items in the PCCoc/Rheum Measuring PCC.

Items	Completely agree % (*n*)^a^	Agree % (*n*)	Disagree % (*n*)	Totally disagree % (*n*)	Missing (*n*)^b^	Mean (*SD*)
1. Welcoming care environment	54.0 (68)	34.1 (43)	10.3 (13)	1.6 (2)	X	2.40 (0.74)
2. Undisturbed conversations	83.3 (105)	15.9 (20)	0.8 (1)	X	X	2.83 (0.40)
3. Equality in meeting	71.4 (90)	27.0 (34)	0.8 (1)	0.8 (1)	X	2.69 (0.53)
4. Confirmed as a person	77.0 (97)	20.6 (26)	1.6 (2)	0.8(1)	X	2.74 (0.52)
5. Opportunity to tell my story	77.8 (98)	18.3 (23)	4.0 (5)	X	X	2.74 (0.52)
6. Understanding my situation	62.1 (77)	33.1 (41)	4.0 (5)	0.8 (1)	2	2.56 (0.61)
7. Experiences are respected	66.4 (83)	30.4 (38)	2.4 (3)	0.8 (1)	1	2.62 (0.58)
8. Self-knowledge is considered	61.0 (75)	30.9 (38)	7.3 (9)	0.8 (1)	3	2.52 (0.67)
9. Problems are taken seriously	79.0 (98)	18.5 (23)	1.6 (2)	0.8 (1)	2	2.76 (0.52)
10. Needs determine care planning	58.9 (73)	35.5 (44)	4.8 (6)	0.8 (1)	2	2.52 (0.63)
11. Agree with a nurse on what to do	66.1 (82)	30.6 (38)	2.4 (3)	0.8 (1)	2	2.62 (0.58)
12. Gain new knowledge	52.8 (65)	43.1 (53)	4.1 (5)	X	3	2.49 (0.58)
13. Strengthened ability to cope	54.8 (68)	39.5 (49)	4.0 (5)	1.6 (2)	2	2.48 (0.66)
14. Coordinated care	70.5 (86)	23.0 (28)	5.7 (7)	0.8 (1)	4	2.63 (0.63)
15. Family participation	60.5 (75)	25.8 (32)	10.5 (13)	3.2 (4)	2	2.44 (0.81)
16. Care follow-up and documentation	73.2 (90)	21.1 (26)	5.7 (7)	X	3	2.67 (0.58)
17. Care responsibility is clear	53.7 (66)	40.7 (50)	4.1 (5)	1.6 (2)	3	2.46 (0.66)
18. Confident nurse contacts	75.2 (94)	20.8 (26)	3.2 (4)	0.8 (1)	1	2.70 (0.579
19. Sufficient time allocated	64.8 (81)	29.6 (37)	5.6 (7)	X	1	2.59 (0.60)
20. Good nurse collaboration	77.6 (97)	19.2 (24)	3.2 (4)	X	1	2.74 (0.51)
21. Information facilitating decisions	59.3 (73)	33.3 (41)	6.5 (8)	0.8 (1)	3	2.51 (0.66)
22. Can influence care	38.3 (46)	45.8 (55)	12.5 (15)	3.3 (4)	6	2.19 (0.78)
23. Personal information documented	63.0 (75)	29.4 (35)	5.9 (7)	1.7 (2)	7	2.54 (0.69)
24. Care information shared as needed	66.1 (80)	29.8 (36)	3.3 (4)	0.8 (1)	5	2.61 (0.60)

*Note.* X = no responses for this alternative; PCCoc/rheum = the Person-Centered Care instrument for outpatient care in rheumatology.

a Column percentage displayed.

b Missing cases are excluded from the analyses.

### Experiences of PCC associated with individual characteristics and HRQoL

There was a significant association (B = 0.214, *p* = .02) between age and sum score for PCC, with a higher likelihood of perceptions of higher PCC with increasing age. Marital status (B = 6.265, *p* = 0.01) was also significantly associated with the sum score for PCC, with a higher likelihood of perceptions of higher PCC for single people (including single, divorced, or widowed). R2 (0.130) indicated a medium explanatory factor of the model (Table 3). The regression analysis showed no significant association between the sum score of the perceived degree of PCC (the PCCoc/rheum) and sex, educational level, having children living at home or having been transplanted during the COVID-19 pandemic. The linear regression analysis for PCC sum score and associations with current global health sum score showed a significant association, with a higher likelihood of higher PCC sum scores with a higher current global health sum score (B = 0.111, *p* = 0.06, CI: 0.03–0.189).

**Table 3.** Results From Multiple Linear Regression Analysis for Associations With Sum Score PCC.

Model	*B*	Std. Error	Beta	*t*	Sig.*	Lower bound	Upper bound
Constant	46.206	5.917		7.809	<.00	34.475	57.937
Age	0.214	0.094	.253	2.283	**.02**	0.028	0.400
Sex (male reference category)	−0.958	1.934	−.046	−0.496	.62	−4.792	2.875
Marital status (married/partner reference category)	6.265	2.395	.240	2.616	**.01**	1.517	11.013
Education (higher education reference category)	−2.930	2.035	−.140	−1.440	.15	−6.964	1.104
Children living at home (yes reference category)	2.443	2.535	.105	0.960	.33	−2.593	7.459
Transplant during COVID-19 (before reference category)	−0.320	2.308	−.013	−0.139	.89	−4.896	4.257

*Note*. Cases with missing values for the independent variables are excluded from the analysis.

* Regression coefficient significant if *p* ≤ .05, significant values in bold.

## Discussion

Our results showed that a large majority of the people who had undergone an allo-HSCT agreed or completely agreed with the fulfillment of person-centeredness for most aspects of the care investigated. The aspects the participants had rated as most person-centered were undisturbed conversations, followed by being taken seriously, having had an opportunity to tell their story, good collaboration with the nurse, being confirmed as a person, equality in the care meeting, and coordinated care. There were, however, lower ratings for, for example, gaining new knowledge in the meeting with the nurse, the patient’s part of the care responsibility being clear, the possibility to influence care, and family participation. Significant associations were seen for age and marital status, with a higher likelihood of reporting higher person-centeredness of care with a higher age and if single.

The high ratings of PCC should be considered bearing in mind that the participants in the study had a fatal illness, which—through treatment and care—they have been cured of, and hence they are grateful for the care. Given the nature of the treatment, the patients are isolated in their room at the ward, which of course can have positive and negative sides, with an up-side being the opportunity to have undisturbed conversations, while a negative side is the isolation experienced. The higher ratings of PCC focused on central elements of a PCC approach—to be seen as a person, to be able to tell their story, and to work in a partnership with healthcare (Ekman et al., 2011). Good collaboration with the nurse is not necessarily the same as partnership in care, and the lower ratings of the possibilities to influence the patients’ own care do indicate that care was not totally given in a partnership, with the patient taking on an informed, active role in shared decision-making. Not being able to influence one’s care can also include the actual medical treatment, which may not be an option for patients in need of an allo-HSCT, where the choice is between a possible cure and a longer life with treatment. A review study about decision-making for or against cancer treatment showed that younger patients were more likely to undergo aggressive treatment to increase survival years. Preference for quality of life without treatment—rather than possible extended length of life—was not influenced by gender, education, religion, having children, marital status, or type of cancer, but patients with better health valued possible lengthening of life through treatment, while those with poorer physical status preferred no treatment for a better quality of life (Shrestha et al., 2019). Covvey et al. (2019) concluded in a review study that barriers to shared decision-making in oncology care from the patient’s perspective are uncertainty or lack of consensus in the treatment decision, concern regarding adverse effects, and poor physician communication. On the other hand, the study showed facilitators were physicians consideration of patient preferences, positive physician actions/behaviors, and use or encouragement of support systems, such as family/friends or people who have had similar experiences. Central for shared decision-making in oncology care was information and communication, and patients who were more involved were also more satisfied with the care (Covvey et al., 2019). Another study stressed the importance of the nurse–patient relationship for care quality and patient autonomy in decision-making about hospital care and revealed the dominance of protective paternalism in decision-making, as opposed to an informed choice model. To enable patient autonomy in decision-making regarding care, an equal distribution of power is needed, in which the professionals are more witnessing, and providing advice, during the health and illness process in the patient and family, rather than adopting the power-skewed approach of today with a paternalistic protective role of the nurse (Molina-Mula & Gallo-Estrada, 2020). Furthermore, our study shows that almost 1 in 10 of the participants did not think that their self-knowledge was considered in care. Influencing the actual treatment can be difficult but participating in one’s care is a necessity in terms of understanding, knowledge, and the opportunity to prepare for self-care after discharge from the hospital. PCC is important to enable patients to prepare for the time before, during, and after hospital care. In a recently published study, nurses working with allo-HSCT reported that due to the very medical focus of allo-HSCT care, nursing became fragmented, and opportunities for PCC, such as care planning or preparing patients for discharge and management of self-care became limited (Holmberg et al., 2023).

Our study showed high ratings of person-centeredness of care; however, almost a third of the participants had PCC sum scores below 59 and about a sixth as low as 20–53. This indicates room for improvement, possibly with a focus on the items with lower ratings—foremost the possibility to influence care and family participation. It may be that the isolation stemming from the nature of the illness and treatment affects family participation in care Our results show no significant associations between ratings of PCC and participants receiving their allo-HSCT during the COVID-19 pandemic, although COVID-19 may have affected family inclusion due to regulations resulting in even further isolation. Furthermore, this study showed significant associations between ratings of PCC and marital status, with a higher likelihood of reporting higher person-centeredness of care if single. A review study found that several studies have tried to involve family members further in care through interventions (Deek et al., 2016), showing positive impacts on readmission rates, emergency department presentations, and anxiety levels, by comparing family-centered interventions with controls. Effective interventions used a family-centered approach, active learning strategies, and transitional care with appropriate follow-up. Family involvement showed positive effects on patient outcomes when informational, instrumental and emotional support, tailored to the patient’s needs, was provided (Deek et al., 2016). Our study showed a higher likelihood of higher PCC ratings with age and if single. Marital status and age impact the life situation, for example, living alone, having a partner and/or children who can be affected by one’s illness, transplantation, and care. The lower ratings of family involvement in care could contribute to a higher likelihood of lower PCC ratings if not single.

In this study, those who had undergone an allo-HSCT 12–36 months prior to the study had on average a medium to highly rated general health, with a mean Global health sum score of 68.5. This is slightly lower than in a healthy general population (Derogar et al., 2012; Nolte et al., 2019), but in line with scores in populations who had undergone an HSCT (Grulke et al., 2012). The impact of allo-HSCT on HRQoL has been shown in previous studies, with patients experiencing lower HRQoL due to many post-allo symptoms and side effects (El-Jawahri et al., 2016; Eriksson et al., 2023; Mosher et al., 2011). Our results showed an association between a higher rating of PCC and higher HRQoL. The connection between PCC and patients’ HRQoL has been investigated in previous studies, indicating more PCC is associated with a higher HRQoL (Brännström & Boman, 2014; Pirhonen et al., 2017).

### Methodological considerations

Self-reporting data can be affected by an external bias caused by social desirability or approval. A way to minimize this is to ensure anonymity and confidentiality. Recall bias can either underestimate or overestimate the true effect or association (Althubaiti, 2016). Patients’ gratitude for the care and potentially being cured of a fatal illness is a risk for desirable answers; therefore, it was deemed preferable to collect data from those who were no longer in care, but there is of course a risk of recall bias instead given the time elapsed since care. Response shift can be seen as well since HRQoL scores were relatively high, despite previous studies showing that people suffer from several symptoms long after transplantation.

This study had a better response rate than would be expected from this type of cross-sectional survey study. The lack of information about the non-responders is to be considered a limitation but was due to anonymity. The PCCoc/rheum instrument is newly developed and was not initially intended for allo-HSCT patients or in-patient care. The phrasing of the questions also makes it difficult to know which part of the care the participants intend when responding, that is, whether they consider all care during their transplant—prior, during, and after. The study has a low number of missing items, indicating the internal validity of the instrument. Cronbach’s alpha for the full scale (24 items) had good results (0.96), indicating that the 24 items measure what was intended. The PCCoc/rheum is deemed viable for use in the context of allo-HSCT, in- and out-patient care, although further psychometric evaluations would strengthen this. Allo-HSCT involves a very specific treatment and care; in addition, the study site is specialized in mainly providing HCT-care. However, the results of this study are considered transferable to other contexts where allo-HSCTs are performed. Despite methodological limitations, this study provides novel and important knowledge about patients’ experiences of PCC, and its associations with individual characteristics and HRQoL, in the context of allo-HSCT.

## Conclusions

The fulfillment of PCC was rated as high, but the results indicate that there is room for improvement regarding the possibility of influencing the care and family members’ involvement. Furthermore, this study reveals differences in reports of care experiences associated with marital status, age, and an association with HRQoL—highlighting the importance of tailoring care to meet the individual needs of patients undergoing an allo-HSCT.
